# Planned early delivery versus expectant management to reduce adverse pregnancy outcomes in pre-eclampsia in a low- and middle-income setting: study protocol for a randomised controlled trial (CRADLE-4 Trial)

**DOI:** 10.1186/s13063-020-04888-w

**Published:** 2020-11-23

**Authors:** Alice Beardmore-Gray, Nicola Vousden, Umesh Charantimath, Geetanjali Katageri, Mrutyunjaya Bellad, Kunda Kapembwa, Sebastian Chinkoyo, Bellington Vwalika, Matthew Clark, Rachael Hunter, Paul Seed, Shivaprasad Goudar, Lucy C. Chappell, Andrew Shennan

**Affiliations:** 1 Department of Women and Children’s Health, School of Life Course Sciences, King’s College London, London, UK; 2 Women’s and Children’s Health Research Unit, KLE Academy of Higher Education and Research, JNMC, Belagavi, Karnataka India; 3 BVV Sangha’s S Nijalingappa Medical College & HSK Hospital and Research Centre, Bagalkot, Karnataka India; 4 Department of Paediatrics, University of Zambia, Lusaka, Zambia; 5 Department of Obstetrics and Gynaecology, Ndola Teaching Hospital, Ndola, Zambia; 6 Department of Obstetrics and Gynaecology, University of Zambia, Lusaka, Zambia; 7 Welbodi Partnership, Ola During Children’s Hospital, Freetown, Sierra Leone; 8 Research Department of Primary Care and Population Health, Royal Free Campus, University College London, London, UK

**Keywords:** Pre-eclampsia, Hypertension, Pregnancy, Perinatal, Global health, Low- and middle-income countries

## Abstract

**Background:**

Pre-eclampsia is a pregnancy complication characterised by high blood pressure and multi-organ dysfunction in the mother. It is a leading contributor to maternal and perinatal mortality, with 99% of these deaths occurring in low- and middle-income countries (LMIC). Whilst clear guidelines exist for management of early-onset (< 34 weeks) and term (≥ 37 weeks) disease, the optimal timing of delivery in pre-eclampsia between 34^+ 0^ and 36^+ 6^ weeks is less clear. In a high-income setting, delivery may improve maternal outcomes without detriment to the baby, but this intervention is yet to be evaluated in LMIC.

**Methods:**

The CRADLE-4 Trial is a non-masked, randomised controlled trial comparing planned early delivery (initiation of delivery within 48 h of randomisation) with routine care (expectant management) in women with pre-eclampsia between 34^+ 0^ and 36^+ 6^ weeks’ gestation in India and Zambia. The primary objective is to establish whether a policy of planned early delivery can reduce adverse maternal outcomes, without increasing severe neonatal morbidity.

**Discussion:**

The World Health Organization recommends delivery for all women with pre-eclampsia from 37 weeks onwards, based on evidence showing clear maternal benefit without increased neonatal risk. Before 34 weeks, watchful waiting is preferred, with delivery recommended only when there is severe maternal or fetal compromise, due to the neonatal risks associated with early preterm delivery. Currently, there is a lack of guidance for clinicians managing women with pre-eclampsia between 34^+ 0^ and 36^+ 6^ weeks. Early delivery benefits the mother but may increase the need for neonatal unit admission in the infant (albeit without serious morbidity at this gestation). On the other hand, waiting to deliver may increase the risk of stillbirth, fetal growth restriction and hypoxic brain injury in the neonate as a result of severe maternal complications. This is especially true for LMIC where there is a higher prevalence of adverse events. The balance of risks and benefits therefore needs to be carefully assessed before making firm recommendations. This is the first trial evaluating the optimal timing of delivery in pre-eclampsia in LMIC, where resources and disease burden are considerably different.

**Trial registration:**

ISRCTN 10672137. Registered on 28 November 2019.

## Background

Pre-eclampsia is a pregnancy-specific disorder which complicates 2–8% of pregnancies worldwide [1] and up to 12% of pregnancies in low- and middle-income countries [2]. Pre-eclampsia is responsible for 76,000 maternal deaths and 500,000 perinatal deaths each year [2] with the overwhelming majority (99%) of these occurring in Sub-Saharan Africa and South Asia [3].

Pre-eclampsia is a multi-system disorder. It arises due to inadequate perfusion of the uteroplacental unit, leading to hypoxic placental tissue and endothelial dysfunction. The resulting systemic vascular inflammation leads to widespread organ involvement in the mother as well as growth restriction and even stillbirth in the fetus [1]. Its clinical course is difficult to predict, and the development of symptoms is usually an indicator of end-stage organ damage. The only definitive management of pre-eclampsia is delivery of the dysfunctional placental unit—thereby ending the pregnancy. Given the progressive and unpredictable nature of the condition, timely intervention and delivery is key.

Delivery at 37 weeks onwards is recommended by the World Health Organization for all women with pre-eclampsia irrespective of disease severity [4]. Prior to 34 weeks (which is an important milestone for fetal lung maturity), expectant management is preferable due to the neonatal risks associated with early preterm birth [4]. Therefore, delivery before 34 weeks’ gestation is usually only initiated if there are signs of severe maternal or fetal compromise.

Guidance on the optimal timing of delivery in late preterm pre-eclampsia (between 34^+ 0^ and 36^+ 6^ weeks’ gestation) is less clear and is likely to be context dependent. In different settings, the risks and benefits of delivery may vary according to the prevalence and character of serious adverse events and the facilities available to manage them.

Currently, a policy of close surveillance is pursued until either 37 weeks’ gestation is reached (at which point delivery is recommended) or an indication for immediate delivery (evidence of severe maternal or fetal compromise) develops. It is likely that planned early delivery would benefit the mother as this is the cure to the disease process; however, this must be balanced against any potential risks associated with late preterm delivery to the neonate.

In high-income settings, previous randomised controlled trials have shown that planned early delivery between 34^+ 0^ and 36^+ 6^ weeks’ gestation in pre-eclampsia reduces the risk of severe complications in the woman [5–7]. An increase in neonatal unit admissions amongst infants in the planned delivery group has been reported, though serious neonatal morbidity remains uncommon at this gestation [5]. Planned early delivery has only been shown to increase respiratory distress syndrome in the neonate when the study population included women with gestational hypertension with a longer time to delivery interval in the usual care arm [6]. This and the fact that antenatal corticosteroid use was less prevalent in this study may explain the difference in neonatal respiratory morbidity between the two arms.

This question is yet to be evaluated in a low- and middle-income setting. Planned early delivery at this gestation may increase risk to the neonate given the lack of neonatal intensive care facilities. In addition, the availability of antenatal corticosteroids and indeed their impact on neonatal outcomes is yet to be fully evaluated in low- and middle-income countries [8, 9]. However, in settings where the disease burden and incidence of serious complications (in particular eclampsia, renal insufficiency, abruption and stillbirth) are related, in part, to inadequate surveillance and delayed intervention, planned early delivery may in fact confer even greater benefit for the woman and the infant to that seen in a high-income setting. Severe disease in this setting implies time to delivery intervals will be shorter, and the benefit of removing maternal harm relatively greater than the risk of immaturity. Given the disproportionate number of maternal and perinatal deaths occurring in low- and middle-income countries, it is imperative that interventions designed to reduce mortality and morbidity are developed and tested within these settings, where their impact may be considerably different.

There is therefore a need to compare a policy of planned early delivery to expectant management for late preterm pre-eclampsia in low- and middle-income settings. This trial aims to establish whether planned early delivery in women with pre-eclampsia between 34^+ 0^ and 36^+ 6^ weeks’ gestation can reduce adverse pregnancy outcomes in India and Zambia.

## Methods/design

### Trial objectives

The aim of this trial is to establish whether planned early delivery in pre-eclampsia between 34^+ 0^ and 36^+ 6^ weeks can reduce adverse pregnancy outcomes compared to expectant management in a low- and middle-income setting.

### Primary objectives

The primary objectives of the study are:

1. To evaluate whether planned early delivery for women with pre-eclampsia between 34^+ 0^ and 36^+ 6^ weeks of gestation can reduce maternal mortality and morbidity based on a composite of outcomes during pregnancy and delivery, until primary hospital discharge.
2. To evaluate the impact of the intervention on short-term neonatal outcomes. These will be assessed based on a composite of stillbirth, neonatal death and neonatal unit admission for > 48 h due to neonatal morbidity, until primary hospital discharge.

### Secondary objectives

The secondary objectives of the study are:

1. To evaluate the impact of the intervention on individual components of the primary outcomes and other secondary short-term outcomes for the mother and baby.
2. To evaluate the impact of the intervention on health resource use and cost.
3. To assess how the intervention influences the experiences of women.
4. To evaluate how the effectiveness of the intervention and its implementation is influenced by external factors (specifically resource availability and health system factors).

### Trial design

- This will be a pragmatic, multicentre, randomised controlled trial of planned delivery versus expectant management in 872 women with pre-eclampsia between 34^+ 0^ and 36^+ 6^ weeks of gestation inclusive.

### Study setting

The trial will be conducted in five tertiary hospitals across India and Zambia, including their referring district healthcare facilities (sites listed on http://www.isrctn.com/ISRCTN10672137). An initial 6-month feasibility study was conducted across the proposed trial sites. This was a mixed-methods study consisting of semi-structured interviews with a cross-section of healthcare providers, focus group discussions with pregnant women and their relatives and a retrospective case notes audit evaluating gestation-specific maternal and neonatal outcomes in women with pre-eclampsia. The results of this feasibility study directly informed the development of the interventional phase protocol.

Recruitment is anticipated to take 22 months based on an assumption that approximately 45 participants will be recruited per month (across all sites), with some allowance for unforeseen events and centres recruiting slower than expected. Daily visits by the research team to the relevant clinical areas at each healthcare facility will ensure that all potentially eligible participants are screened. In addition to this, key personnel at each of the referring healthcare facilities will be provided with a basic mobile phone and airtime in order to facilitate referrals of potentially eligible participants. The development of culturally appropriate trial materials for both participants and key members of their household will help to engage and inform potential participants. Dissemination of trial posters and flowcharts will ensure that clinical staff are well informed and aware of trial procedures. If necessary, additional strategies to boost trial recruitment (such as additional sites or small financial incentives for clinical staff will be considered).

### Selection and withdrawal of participants

#### Inclusion criteria

Women who meet the following criteria will be eligible for enrolment into the study:

- Able to give valid written, informed consent
- Viable ongoing pregnancy at time of recruitment
- Clinical diagnosis of pre-eclampsia confirmed by the obstetric team: must fulfil minimum criteria of hypertension and proteinuria after 20 weeks’ gestation. Hypertension will be defined as a systolic blood pressure of ≥ 140 mmHg and/or a diastolic blood pressure of ≥ 90 mmHg (or on anti-hypertensive drug at enrolment). Proteinuria will be defined as a ‘positive’ (≥ 1 + protein) urine dipstick result [10].
- Gestational age between 34^+ 0^ and 36^+ 6^ confirmed by a doctor (as determined by known last menstrual period date validated by early or late ultrasound scan if available)

Women with any other co-morbidity (including pre-existing hypertension, diabetes, and HIV) or having had a previous caesarean section or with the fetus in any position will be eligible. Women with multi-fetal pregnancy will also be eligible.

#### Exclusion criteria

Women will be excluded from participation in the study if a decision has already been made to deliver within the next 48 h.

### Recruitment, eligibility and consent

Members of the research team will provide a full verbal explanation and written description (in the relevant local language) to women who meet the inclusion criteria (as above). Additionally, participant information videos in local languages have been developed to aid comprehension amongst both trial participants and their relatives. The woman will be given sufficient time to consider the information and to decide whether she will participate in the trial. Written informed consent will be sought from the woman and taken by an appropriately trained member of the research team.

### Study periods

**Fig. 1:**
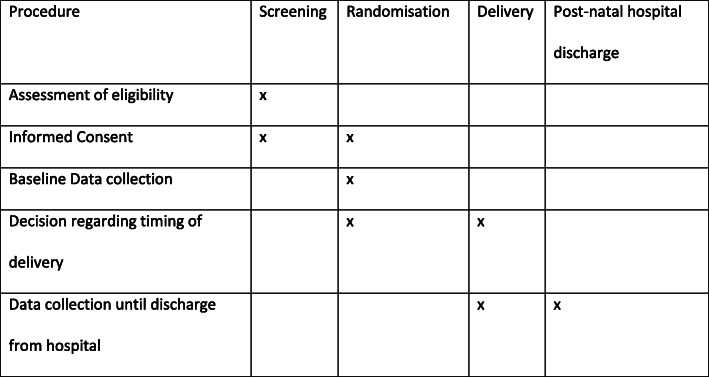
Schedule of participant enrolment, interventions and assessment in the trial (SPIRIT figure)

A woman’s participation in the study may be from 34 weeks’ gestation until primary discharge of the woman and her baby after birth (Fig. 1). Long-term follow-up will be considered by obtaining permission to contact participants later, but only after further ethical approval and governance has been ascertained. Both the maternal and neonatal short-term outcomes will be collected quickly as the time period from randomisation to outcome collection will not exceed 14 weeks (participants will be followed up until primary discharge of mother and baby post-delivery) and in many cases will be less. Outcome collection will end 42 days after the final participant has been recruited (or sooner if primary discharge of mother and baby occurs before this endpoint).

### Withdrawal of participants

At all stages, it will be made clear to the woman that she is free to withdraw from the trial at any time without the need to provide any reason or explanation. Participants will be made aware that this decision will have no impact on any aspect of their continuing care. For a woman allocated to the expectant management group, if clinical needs dictate delivery prior to 37 weeks’ gestation based on local criteria, this will not constitute withdrawal from the trial allocation. For a woman allocated to the planned delivery group, if the woman should decide that she does not wish to proceed with the planned delivery and instead chooses to be monitored by her attending clinician, this will not constitute withdrawal from the study.

### Assessment of outcomes

Outcomes will be recorded on the web-based database after a review of case notes by trained members of the research team. This will be done contemporaneously and completed no later than 24 h after the mother and baby have been discharged. Confirmation of maternal and neonatal outcome data will be undertaken with an additional sign-off by the site’s principal investigator for each participant and constant communication with the relevant clinical teams.

### Co-primary outcomes

#### Primary short-term maternal outcome

Primary short-term maternal outcome will include maternal mortality and morbidity based on the miniPIERS composite [11] (see Table 1 for full list) of adverse maternal outcomes (with the addition of severe hypertension) during pregnancy and delivery until primary hospital discharge.

**Table 1:** Full definitions of individual components of the primary short-term maternal outcome

Outcome	Definition
Mortality	Maternal death occurring before primary discharge from hospital
Hepatic dysfunction	Elevated liver enzymes (alanine transaminase or aspartate transaminase ≥ 70 IU/L)
Hepatic hematoma or rupture	Blood collection under the hepatic capsule as confirmed by ultrasound or laparotomy
Glasgow coma score < 13	Based on GCS scoring system [12]
Stroke	Acute neurological event with deficits lasting longer than 48 h
Cortical blindness	Loss of visual acuity in the presence of intact pupillary response to light
Reversible ischaemic neurologic deficit (RIND)	Cerebral ischaemia lasting longer than 24 h but less than 48 h revealed through clinical examination
Retinal detachment	Separation of the inner layers of the retina from the underlying retinal pigment epithelium (RPE; choroid) and is diagnosed by ophthalmological exam
Acute renal insufficiency	For women with an underlying history of renal disease: defined as creatinine > 200 μM; for patients with no underlying renal disease: defined as creatinine > 150 μM
Dialysis	Including haemodialysis and peritoneal dialysis
Postpartum haemorrhage (PPH) requiring transfusion or hysterectomy	Occurrence of PPH that required transfusion or hysterectomy
Placental abruption	Any occurrence of abruption diagnosed clinically or based on placental pathology report
Platelet count < 50,000 without blood transfusion	Measurement of platelet count recorded as less than 50,000 without patient being given a blood transfusion
Transfusion of blood products	Includes transfusion of any units of blood products: fresh frozen plasma (FFP), platelets, red blood cells (RBCs), cryoprecipitate (cryo) or whole blood. Includes request for transfusion even if products unavailable at time of request.
Positive inotropic support	The use of vasopressors to maintain a systolic blood pressure > 90 mmHg or mean arterial pressure > 70 mmHg
Myocardial ischaemia/infarction	ECG changes (ST segment elevation or depression) with ischaemic symptoms with or without typical enzyme changes
Eclampsia	Any episode of seizure antepartum, intrapartum or before postpartum discharge as follow-up beyond discharge is not possible
Require > 50% oxygen for greater than 1 h	Oxygen given at greater than 50% concentration based on local criteria for longer than 1 h
Intubation other than for Caesarean section	Intubation may be by endotracheal tube insertion or continuous positive airway pressure
Severe breathing difficulty	Suspected pulmonary oedema where X-ray confirmation is unavailable may be diagnosed by presence of chest pain or dyspnoea, crackles in the lungs and SaO_2_ < 90%
Pulmonary oedema	Clinical diagnosis with X-ray confirmation or requirement of diuretic treatment and SaO_2_ < 95%
Severe hypertension	Systolic blood pressure of ≥ 160 mmHg between randomisation and post-delivery discharge

#### Primary short-term perinatal outcome

Primary short-term perinatal outcome will include composite of one or more of antenatal/intrapartum stillbirth or neonatal death (but not deaths due to congenital anomalies) or neonatal unit admissions > 48 h due to neonatal morbidity (necessitating admission to the neonatal unit according to local guidelines) until primary hospital discharge.

### Secondary outcomes

Secondary maternal outcomes will include assessment of:

- Individual components of the primary outcome
- Mode of onset of birth (spontaneous, induced or pre-labour caesarean section)
- Primary indication for delivery in both arms
- Intensive care unit admission
- Length of stay in hospital (prior to delivery and after delivery)
- Time from randomisation to delivery (process outcome)
- Use of magnesium sulfate
- Use of antenatal corticosteroids for fetal lung maturity
- Use of anti-hypertensive medications

Secondary perinatal outcomes will include assessment of:

- Individual components of the primary outcome
- Mode of delivery (vaginal vs. all others)
- Gestational age at delivery
- Birthweight
- Birthweight centile
- Admissions to neonatal unit (and primary indication)
- Total number of nights in hospital and number of nights in each level of care for babies admitted
- Sepsis—with evidence of confirmed infection
- Course of antibiotics given for possible serious bacterial infection (according to the World Health Organization’s Integrated Management of Childhood Illness (IMCI) guidelines) [13]
- Apgar score at 5 and 10 min post birth
- Need for neonatal resuscitation
- Hypoxic ischaemic encephalopathy and grade
- Neonatal seizures requiring anti-convulsants
- Respiratory distress syndrome
- Supplementary oxygen and duration required
- Use of continuous positive airway pressure ventilation and duration required
- Invasive ventilation support and duration required
- Administration of surfactant
- Hypoglycaemia (< 2.6 mmol) requiring intervention
- Hypothermia (temperature < 36.5 °C)
- Neonatal jaundice requiring phototherapy
- Necrotising enterocolitis (diagnosed at surgery or resulting in death)
- Nasogastric feeding required and indication
- Exclusively breast-fed at discharge from hospital

### Trial procedures

#### Informed consent

Written consent will be sought from the woman only after she has been given a full verbal explanation and written description of the trial (via the participant information leaflet, in her preferred language). The local research team at each site are fluent in English and the relevant local languages spoken by the majority of the population across the trial sites (Bemba and Nyanja at the Zambian sites, Kannada at the Indian sites). The participant information leaflet will be read aloud to women who are unable to read it themselves. Partners and relatives will be included in the discussion but may not consent on the woman’s behalf. Additionally, three short video clips addressing key topics (pre-eclampsia, trial participation and the neonatal unit) will be made available to all potentially eligible participants, particularly those with limited literacy. Written informed consent will be given using an informed consent form, completed, signed (thumbprints also accepted) and dated by the woman and signed by the member of the research team who obtained informed consent. After written informed consent has been obtained, a member of the research team will enter the baseline maternal details onto the online database and perform randomisation, communicating the results directly to the woman and her clinical team.

Antenatal, intrapartum and postpartum care will be in accordance with local guidelines and capacity at each site. Delivery will typically be through induction according to local protocol (most commonly oral or vaginal administration of misoprostol). The schedule of care for each group will be as follows:

#### Intervention (planned delivery) group

The intervention is planned delivery, to be undertaken as soon as feasible (aimed to be commenced within 48 h) after randomisation. Use of antenatal corticosteroids for fetal lung maturity will be at the discretion of the clinician, in accordance with local guidelines (confirmed as readily available across all facilities). Postnatal care will be in accordance with local protocols and guidelines.

#### Control (expectant management) group

Expectant management involves close monitoring of the maternal and fetal condition until the woman reaches 37 weeks, or a crisis develops necessitating delivery. Delivery is recommended if the woman develops severe pre-eclampsia. This is in accordance with the World Health Organization guidelines [4] which are followed at all of the proposed trial sites.

#### Time of delivery—adherence to protocol

Following randomisation to either the planned delivery group or expectant management group, the time of onset of planned delivery (first method for induction of labour or time of planned caesarean section along with the indication) or onset of spontaneous labour will be recorded for all women. This will enable the monitoring of adherence to protocol for both study groups to be reviewed and protocol deviations to be identified and investigated.

### Sample size

The sample size for the CRADLE 4 study is calculated on the ability to detect a clinically important reduction in the primary maternal outcome: a short-term composite based on the presence of one or more of 22 maternal morbidities. Based on data acquired at the sites prior to start of the main trial, we anticipate an event rate of 80% for the primary maternal outcome in the expectant management arm. We have calculated that a sample size of 558 would provide 90% power to detect a 15% relative risk reduction. If the trial is recruiting well, we will continue to recruit 872 participants which would give 90% power to detect a 12.5% relative risk reduction and greater precision to detect secondary outcomes. The data monitoring committee (DMC) will review the primary event rate and usual safety data and make a recommendation to continue or stop. A one-sided non-inferiority analysis is planned for the primary neonatal composite. Our data acquired at the sites prior to starting the main trial showed an event rate of 24% for the primary neonatal outcome. Complete data on 480 women (240 per group) are required for 90% power to exclude a difference against planned delivery of 10% or more. To exclude a difference of 7.5%, 852 women (426 per group) are needed. The calculation uses a one-sided significance test and confidence interval and assumes that the true event rate is 24%. This is in line with the planned sample size as detailed above.

### Randomisation

Randomisation will be managed by a secure web-based randomisation facility hosted by MedSciNet. The allocation ratio of intervention (planned early delivery) to control (expectant management) will be 1:1. Participants will be stratified by centre and minimised by parity (0 or ≥ 1), single/multi-fetal pregnancy (singleton or multi-fetal) and gestational age (34^+ 0^–34^+ 6^, 35^+ 0^–35^+ 6^, 36^+ 0^–36^+ 6^) at randomisation. MedSciNet will write the randomisation programme and hold the allocation code. Following randomisation, a clinician will then arrange for delivery or ongoing expectant management as the randomisation indicates.

### Masking

Due to the nature of this study, masking of clinicians, nursing staff and participants is not possible. In view of arrangements for the conduct of the trial at these sites, it is not feasible to arrange for a separate team of outcome assessors masked to intervention allocation. Data analysis will be conducted masked to group allocation.

### Data collection

Much of the outcome data for this trial are routinely recorded clinical items that can be obtained from the clinical notes. No additional blood or tissue samples are required for this study.

Outcomes will be recorded prospectively using case report forms (CRFs). When possible, online versions will be used (eCRFs) and outcomes therefore recorded directly on the trial database. If, due to power shortages or lack of internet connectivity, this is not feasible, paper case report forms will be used, and data then directly transcribed into the database.

### Assessment of safety

The DMC will ensure the wellbeing of study participants and will periodically review study progress and outcomes as well as reports of unexpected serious adverse events (SAEs). The DMC will, if appropriate, make recommendations regarding continuance of the study or modification of the study protocol.

#### Adverse events

An adverse event is any untoward medical occurrence in a participant, which does not necessarily have to have a causal relationship with this intervention. Due to the high incidence of adverse events routinely expected in this patient population, only those adverse events identified as serious will be recorded for the trial.

#### Serious adverse events

A serious adverse event is any untoward medical occurrence that:

- Results in death
- Is life-threatening
- Requires participant hospitalisation or prolongation of existing hospitalisation
- Results in persistent or significant disability/incapacity

#### Expected SAEs

Expected SAEs are those events which are expected in the patient population or as a result of the routine care/treatment of a patient.

The following events are expected in women with pre-eclampsia and their infants and will be recorded as part of outcome collection (during a woman’s participation in the trial—from randomisation until primary hospital discharge of either mother or baby) but do not require reporting as SAEs.

##### Expected maternal SAEs

- Hepatic dysfunction
- Hepatic haematoma or rupture
- Coma/impaired consciousness (Glasgow coma score < 13)
- Maternal stroke
- Cortical blindness
- Reversible ischaemic neurological deficit
- Retinal detachment
- Acute renal insufficiency or failure
- Postpartum haemorrhage requiring transfusion or hysterectomy
- Placental abruption
- Platelet count < 50,000
- Severe uncontrolled hypertension
- Myocardial ischaemia/infarction
- Eclampsia
- Severe breathing difficulty
- Pulmonary oedema
- Sepsis
- Venous thrombo-embolism
- Admission to hospital for pregnancy and any related pregnancy complications
- Admission to ITU for pregnancy and any related pregnancy complications
- Any pregnancy-related complication requiring surgical management

##### Expected infant SAEs

- Congenital anomaly
- Low birth weight
- Requirement for supplemental oxygen or ventilation support
- Sepsis confirmed by positive cerebrospinal fluid or blood cultures
- Necrotising enterocolitis
- Seizures
- Hypoxic ischaemic encephalopathy
- Hypoglycaemia
- Admission to neonatal unit for any indication

#### Unexpected SAEs

An unexpected SAE is any event that meets the definition of a SAE and is not detailed in the list above as expected.

The following events, whilst not entirely unexpected in this population, are nevertheless serious enough that they should be reported. However, we anticipate that these will be more related to the disease process in this setting and not directly related to the intervention. With this in mind, they will be aggregated and reviewed on a 3-monthly basis by the DMC.

- Maternal death
- Neonatal death
- Antepartum or intrapartum stillbirth

#### Safety reporting procedures

All SAEs (described above) will be recorded from randomisation to postnatal discharge from hospital of mother and baby. Unexpected SAEs for both the mother and infant will be recorded and reported to the DMC as described above. Details of the SAE should be recorded on an SAE form (either electronically via the study database or in paper format). Paper forms will be emailed to the trial coordinating team. An SAE occurring to a participant will be reported to the research ethics committee that gave a favourable opinion of the study where in the opinion of the principal investigator the event was ‘related’ (resulted from administration of any of the research procedures) and ‘unexpected’ in relation to those procedures. Reports of related and unexpected SAEs will be submitted within 15 working days of the principal investigator becoming aware of the event, using the health research authority (HRA) report of serious adverse event form. All reported SAEs will be reviewed by the DMC at regular intervals throughout the study. The principal investigator will inform all investigators concerned of relevant information that could adversely affect the safety of participants.

### Data monitoring and auditing

The site research team will be responsible for the day-to-day smooth running of the trial at a recruiting site. The central trial research team will monitor recruitment against targets, provide staff education and training and monitor the completeness and quality of collected data. The study monitor will perform regular visits to all recruiting centres and will verify the source data for selected participants during these visits.

### Statistical analysis

The primary analysis for all maternal outcomes will be by the intention to treat principle with participants analysed in the groups to which they are assigned regardless of deviation from the protocol or intervention received. We will analyse the difference between arms in the randomisation to delivery interval (3 monthly) to ensure intervention compliance. Women in the expectant management arm will frequently be delivered prior to 37 weeks of gestation due to clinical need and this will not be considered a protocol deviation.

The primary analysis for all perinatal and infant outcomes will be both an intention to treat and a per-protocol analysis, since the hypothesis under examination for these outcomes is a non-inferiority hypothesis. The per-protocol analysis will exclude babies of women who do not receive the allocated intervention as per protocol and will be further defined in the statistical analysis plan.

All outcomes will be analysed adjusting for minimisation factors at randomisation where possible [14]. Where possible, continuous outcomes will be adjusted for baseline measurements of the same variable [15]. Binary outcomes will be analysed using log binomial regression models. Results will be presented as adjusted risk ratios with associated confidence intervals (CI). If the model does not converge, logistic regression with robust variance estimation will be used [16]. Continuous outcomes will be analysed using linear regression models. Results will be presented as differences in means with associated CIs. 95% CIs will be presented for all primary outcomes and 99% CIs for secondary outcomes.

For the analysis of perinatal outcomes, we will treat all infants (singletons or multiples) separately, adjusting standard errors for clustering by mother. Pre-specified subgroup analyses will be undertaken for gestation at randomisation (test for trend) and for single vs. multi-fetal pregnancy, country and region (with a region being tertiary centre and referring healthcare facilities). The consistency of the effect of planned delivery vs. expectant management across subgroups will be assessed using a likelihood ratio test for interaction. Loss to follow-up is expected to be about 5% for the short-term outcomes.

A secondary per-protocol analysis will look at the primary outcomes according to the treatment actually received and time of randomisation.

The primary maternal outcome is maternal mortality and morbidity based on miniPIERS [11] plus severe hypertension (Table 1) during pregnancy or before hospital discharge. The maternal mortality and morbidity component of the primary outcome will be reported separately, as will the severe hypertension component. Additionally, a maternal mortality and morbidity composite of components detected by a clinical diagnosis only will be reported separately (outlined in further detail in the statistical analysis plan).

Health care resource use will include information collected on the management of pre-eclampsia, maternal hospital length of stay related to pre-eclampsia and delivery, maternal intensive care unit admissions and perinatal neonatal unit admissions and hospital length of stay. Health care resource use will be costed using published sources and will be reported in United States Dollars (USD); costs will be reported in local currencies where possible. Mode of onset and mode of delivery will also be included in the costing. Means and standard deviations will be reported for health care resource items and costs. Linear regression and bootstrapping will be used to calculate the difference between treatment groups and 95% confidence intervals, adjusting for minimisation factors at randomisation.

### End of trial

The end of the intervention phase will be when the last participating mother and infant have been discharged from hospital, or 42 days after the final participant has been recruited (whichever occurs sooner). For regulatory purposes, the end of the trial is defined as the date when the study database is locked. An end of study declaration will be made to the approving research ethics committees within 3 months of this date.

### Early cessation

In the light of interim data and other evidence from relevant studies, the DMC will inform the trial steering committee (TSC) if, in its view, there is proof beyond reasonable doubt that the data indicate that the trial should be terminated. A decision to inform the TSC of such a finding will in part be based on statistical considerations.

### Evaluation of women’s experiences

A purposeful sample of participants will be approached for consent to a qualitative interview exploring their experience of the trial intervention (or usual care arm).

### Evaluation of implementation

The impact of external factors (specifically resource availability and health system factors) on the effectiveness of the implementation of the intervention will be assessed by conducting an audit of key resources available at each participating healthcare facility at regular (6 monthly) intervals during the trial, which will be reported using descriptive statistics. A subgroup analysis of the main trial results by site will identify any meaningful variations by site, which may be influenced by local resource availability.

### Data handling

Anonymised data be will collected by the local research team under the supervision of the trial coordinator.

When possible, all anonymised data will be directly entered onto a secure, online database (MedSciNet). If the low-resource nature of the environments where we will be collecting the data means this is not possible, the local research team will be trained to accurately transfer any paper-based data onto MedSciNet, whilst maintaining confidentiality always.

Consent forms and source data where paper based will be kept in files in secure areas at each central site. Only healthcare providers involved in trial participants’ care, research assistants, the local trial coordinator and the UK-based trial manager will have access to these. All paper documents will be stored securely and kept in confidence in compliance with the UK Data Protection Act 1998.

All data entered on the MedSciNet database in each facility will be automatically stored and backed up. Collection and storage of clinical data in the database will be governed by the UK Data Protection Act 1998. All participants will be given a unique trial identifier and no personal information will be entered into the clinical trial database or sample database. Personal contact information will be held on a local database kept in a locked environment, after gaining written informed consent from trial participants.

All MedSciNet data is stored on high-capacity servers that are operated by an external company. Servers are stored in locked rooms, with system monitoring 24 × 7, physical surveillance and surveillance cameras. A tape backup system is used for backing up the database.

The MedSciNet database will remain live for 1 year following completion of the main trial. A copy of this will then be kept on the KCL server for 20 years following the trial completion date, in accordance with the KCL Data retention schedule.

## Discussion

Management of late preterm pre-eclampsia remains a challenging clinical scenario for clinicians around the world. Current evidence does not address those populations and contexts where the primary disease burden of pre-eclampsia lies. Whilst early-onset pre-eclampsia (before 34 weeks’ gestation) is typically regarded as a more ‘severe’ phenotype of the condition, pre-eclampsia at 34 weeks’ gestation onwards is responsible for significant maternal and perinatal morbidity [17]. This is particularly true in low-resource settings where delays in seeking appropriate care and suboptimal quality of care contribute to high rates of maternal and perinatal mortality [18]. Planned early delivery beyond 34 weeks has the potential to reduce serious maternal complications (such as stroke, eclampsia and death) as well as poor perinatal outcomes (such as severe growth restriction and stillbirth). Designing a trial protocol to evaluate this research question in a robust manner, whilst taking into consideration the reality of the trial environment, is challenging and highlights many of the wider barriers to maternal health in low- and middle-income countries. The feasibility phase identified several key issues which informed the design of the main trial protocol, for example, a lack of availability of first trimester ultrasound scanning impacting upon gestational age assessment and lack of laboratory reagents for performing routine kidney and liver function tests. Diagnostic criteria for pre-eclampsia and outcome definitions required adapting to suit the local context, taking into account limited diagnostic resources (e.g. radiology services) and facilities (e.g. neonatal intensive care). Our intervention, if shown to be beneficial, must be reproducible and feasible to implement within a real-world scenario. The inclusion of two diverse countries (India and Zambia) will produce results that are generalisable to similar settings. Furthermore, ensuring that the trial protocol and procedures reflect the reality of maternity care in a low- and middle-income setting is essential in order to produce findings that will be of importance to local, national and international policy makers.

## Trial status

The current CRADLE-4 protocol is version 1.1 (14 November 2019). The trial opened to recruitment on 16 December 2019. The first participant was recruited on 19 December 2019. All trials sites were open by 24 January 2020. Recruitment is ongoing. We anticipate recruitment will be complete by 31 August 2021.

## Supplementary information

**Additional file 1.** SPIRIT 2013 Checklist: Recommended items to address in a clinical trial protocol and related documents.

**Additional file 2.** Model consent form (English version).

**Additional file 3.** Model participant information leaflet (English version).

**Additional file 4.** Statistical Analysis Plan.

## Data Availability

King’s College London will coordinate dissemination of the results from this trial. All publications using data from this trial to undertake original analyses will be submitted to the Trial Steering Committee for review before release. The research will be published in high-impact, peer-reviewed, scientific journals. More general dissemination of the results will be achieved through publication of summary findings. There are no commercial or intellectual rights issues that would delay publication of results. A writing committee drawn from the co-investigators, trial coordinators and others substantially involved in execution, analysis and interpretation will be named authors on the principal publications arising from the trial provided they meet the authorship criteria used by most high-impact peer-reviewed journals (see http://www.icmje.org). No external professional writers will be used. Local principal investigators will be named formally as collaborators on the publication; other trial personnel with significant input to the running of the trial will be named in the Acknowledgements in publications. The Chief Investigator will nominate and agree appropriate authorship on all publications prior to commencement of writing. Participants will be sent a summary of trial publications if they wish, with a reference to the final paper. A copy of the journal article will be made available to them on request from the chief investigator. Given that the majority of participants in this trial will not speak English as a first language and may have limited literacy and computer literacy skills, extensive efforts will be made to disseminate key findings in local languages via community meetings at times convenient to trial participants and their families. To target the clinical community, the results of this research will be disseminated at conventional academic platforms, including presentations at prominent national and international conferences. Requests for the final dataset can be made through the chief investigator in accordance with the data-sharing policies of King’s College London, with input from the co-investigator group where applicable.

## Abbreviations

LMIC: Low- and middle-income countries
HIV: Human immunodeficiency virus
DMC: Data monitoring committee
TSC: Trial steering committee
SAE: Serious adverse event
CI: Confidence interval
ITU: Intensive care unit
PI: Principal investigator
Co-I: Co-investigator
PMG: Project management group
HRA: Health research authority
KCL: King’s College London
REC: Research ethics committee
