# Supplementary material for: Planned early delivery versus expectant management to reduce adverse pregnancy outcomes in pre-eclampsia in a low- and middle-income setting: study protocol for a randomised controlled trial (CRADLE-4 Trial)
Source: Trials. 2020 Nov 23;21:960. doi: 10.1186/s13063-020-04888-w (PMC7684962; doi:10.1186/s13063-020-04888-w)
Supplement: Supplementary file 2 — Additional file 2. Model consent form (English version). [file 13063_2020_4888_MOESM2_ESM.docx]

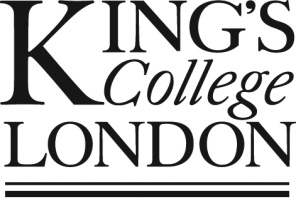
**Consent Form**

**Title of the Study: The CRADLE-4 Trial - Planned early delivery versus expectant management to reduce adverse pregnancy outcomes in pre-eclampsia in a low and middle-income setting.**

Thank you for considering taking part in this research.

The person organising the research must explain the project to you before you agree to take part.

If you have any questions, please ask them before you sign this form.

**Please mark √**

You will be given a copy of this Consent Form to keep.

**I understand that by marking the box next to each statement I am giving my consent and agreeing with each statement. I understand if I leave the box blank it means I do not agree or give my consent which means I may not be eligible to take part in the study.**

**Please mark √**

1. I have read (or had read to me) the information sheet provided. I have asked the researcher any questions that I have, and they have answered them.
2. I agree to take part in this study. I understand I can change my mind at any point and withdraw from the study (up until 31st August 2021) if I want to.
3. I understand that by taking part in this study I may be randomly allocated to either planned early birth or watchful waiting
4. I consent to my personal information being used for the purposes of the study (as explained in the information sheet). I understand my information will be protected by UK data protection laws.
5. I understand that my information may be reviewed by responsible individuals who are monitoring the trial.
6. I understand that my information will be kept confidential and will be anonymised. I understand it will not be possible to identify me or my baby in any research reports.
7. I agree that the research team may use my data for future research if ethical approval from a research committee is gained.
8. I understand that the information I have submitted will be published as a report in a medical journal.
9. I agree that the research team may contact me if necessary (by phone) as part of the research study.

**Name of participant Date Signature or Thumbprint**

**Name of researcher Date Signature**
