# Supplementary material for: Planned early delivery versus expectant management to reduce adverse pregnancy outcomes in pre-eclampsia in a low- and middle-income setting: study protocol for a randomised controlled trial (CRADLE-4 Trial)
Source: Trials. 2020 Nov 23;21:960. doi: 10.1186/s13063-020-04888-w (PMC7684962; doi:10.1186/s13063-020-04888-w)
Supplement: Supplementary file 3 — Additional file 3. Model participant information leaflet (English version). [file 13063_2020_4888_MOESM3_ESM.docx]

| **Who should I contact for further information?**  *Relevant site details provided here* |  |  | **THE CRADLE-4 TRIAL: Can planned early birth in pre-eclampsia (high BP in pregnancy) reduce adverse pregnancy outcomes?**  PARTICIPANT INFORMATION LEAFLET  VERSION 1.1. 2019_10_10  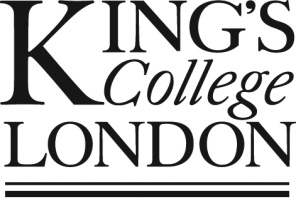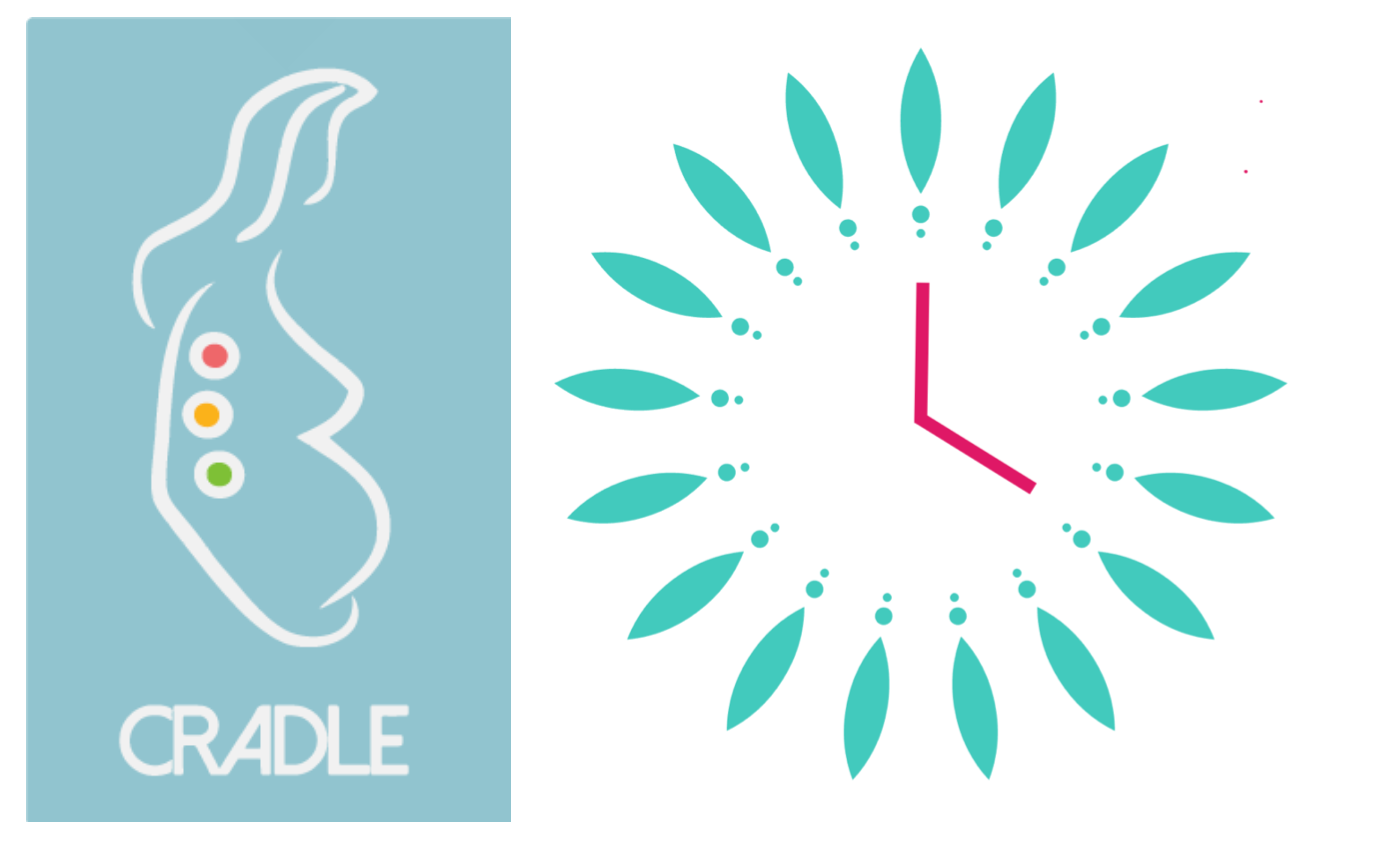 |
| --- | --- | --- | --- |

| **Who are we?**  We are a team of researchers aiming to improve care for women with high blood pressure in pregnancy. We would like to invite you to take part in this research project. Before you decide whether you want to take part, it is important for you to understand why the research is being done and what your participation will involve.  Please take time to read the following information and discuss it with others if you wish. Please ask a member of the research team if there is anything that is not clear or if you would like more information.  **What is the purpose of the study?**  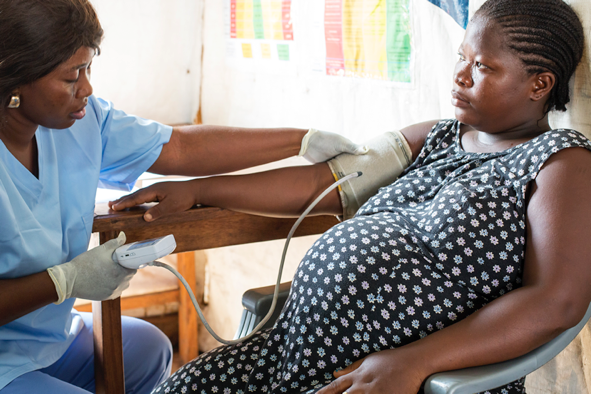  Pre-eclampsia is a pregnancy complication. It is associated with high blood pressure and protein in the urine. It can be a serious condition for both mother and baby.  For example, it can cause very high blood pressure and in severe cases it can cause fits, stroke and sometimes even death.  Babies whose mothers have pre-eclampsia are smaller and are more likely to be born early. In severe cases pre-eclampsia may cause babies to be stillborn (intra-uterine fetal death).  The cause of pre-eclampsia is not known. However, we know that the condition only improves once the baby is delivered.  Once a woman with pre-eclampsia has completed 37 weeks (8 ½ months) of pregnancy it is recommended that she is delivered. Her labour is normally started by induction (using medicines to start labour), between 37 and 38 weeks of |  |  | **How will you manage the information collected about me and my baby?**  If you take part in the study we will collect some personal information. This will only be used by members of the research team if they need to contact you. This information will be kept confidential. This means that only members of the research team will have access to it, and it will not be shared with anybody else.  Any other information collected about you and your baby will be anonymised. This means that you will be given a Study ID number. We will not use your name. It will not be possible to identify you or your baby from these records. The information will be kept on a secure computer. Only members of the research team will have access to it.  The data will be protected according to UK Data Protection Laws.  **What will happen to the results of the study?**  At the end of the study, the results will be analysed and published in scientific journals and presented at scientific conferences. You and your baby will not be identified in any report or publication about the study.  **How is the project being funded and organised?**  This study is being funded by the Medical Research Council in the UK and the Department of Biotechnology in India. The study is organised by King's College London in collaboration with the University of Zambia and the KLE Academy of Higher Education and Research, India.  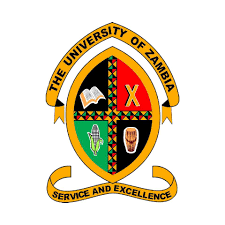  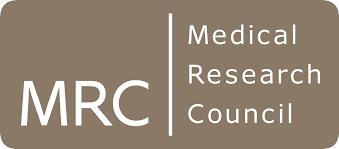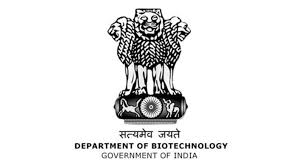 |
| --- | --- | --- | --- |
|  |  |  |  |
|  |  |  |  |
| 1 |  |  | **6** |

1

| If you are in the watchful waiting (expectant) group:  If you are in this group your doctors will be monitoring you and your baby. During your monitoring, your doctors will recommend delivery if they are concerned about either you or your baby’s condition. If this happens they will start your labour early, meaning they will offer you an induction of labour (giving medicines to start your labour). When women have pre-eclampsia, sometimes their babies do not get enough nutrients for them to grow properly and in addition the women can become unwell quickly. If this happens to you, you may need an emergency delivery (either by induction or by caesarean). This may be stressful for you and your baby.  We don't know whether it is better to be in the planned early birth group or the watchful waiting group. This is why we are doing this research.  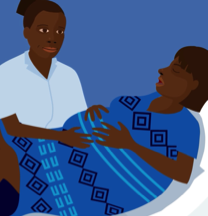**What if I change my mind about taking part?**  You can leave the study at any time, up until 31st August 2021. We will ask you if we may use the information collected about you so far in the analysis of the study. We may also ask if you are happy for the researchers to continue to collect information about the health of you and your baby until you are discharged from hospital However, this is entirely voluntary and you may choose to withdraw all the information collected about you and your baby (up until 31st August 2021) if you wish.  If you decide you no longer want to take part in the study, this will not affect the quality of the routine care provided to you and your baby for the remainder of your pregnancy. |  |  | pregnancy (around 8 ½ months). At this time most babies are fully developed and ready to be born.  But, because pre-eclampsia can be serious and the mother and her baby might become unwell suddenly, it may be better for women with pre-eclampsia to deliver their babies earlier.  This study aims to find out whether, in women with pre-eclampsia between 34 and 37 weeks of pregnancy (around 7 ½ to 8 ½ months), planned early birth can reduce complications for the mother and her baby, compared to waiting until 37 weeks (about 8 ½ months) or until a serious problem occurs before this time.  This study may help improve the care of women with pre-eclampsia in the future.  **Why have I been invited to take part?**  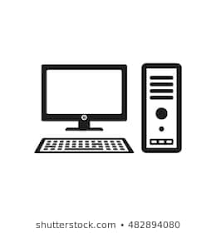You have been invited to take part in this study because you have pre-eclampsia, but your condition does not require that your baby be delivered immediately.  **What will happen if I take part?**  If you decide you would like to take part we will ask you to sign a consent form.  Details about you and your pregnancy will be put into a computer which will randomly allocate you to either the planned early birth group or the watchful waiting group. Random allocation means that you cannot choose which group you want to be in, and neither can your doctor. The computer will randomly allocate you - you will have a 50/50 chance of being in either group.  If you are allocated to the planned early birth group then your labour will be started by your doctor within two days. This is called an induction of labour (using medicines to start your labour). Your doctor might give you some steroid injections to help your baby's lungs mature. |
| --- | --- | --- | --- |
| 5 |  |  | 2 |

| If you don’t go into labour (get labour pains), your doctor may deliver you in other ways, according to the hospital's protocol.  If you are allocated to the watchful waiting group then your doctors will look after you according to their routine protocol. This means you will be admitted to hospital and they will monitor you and your baby.  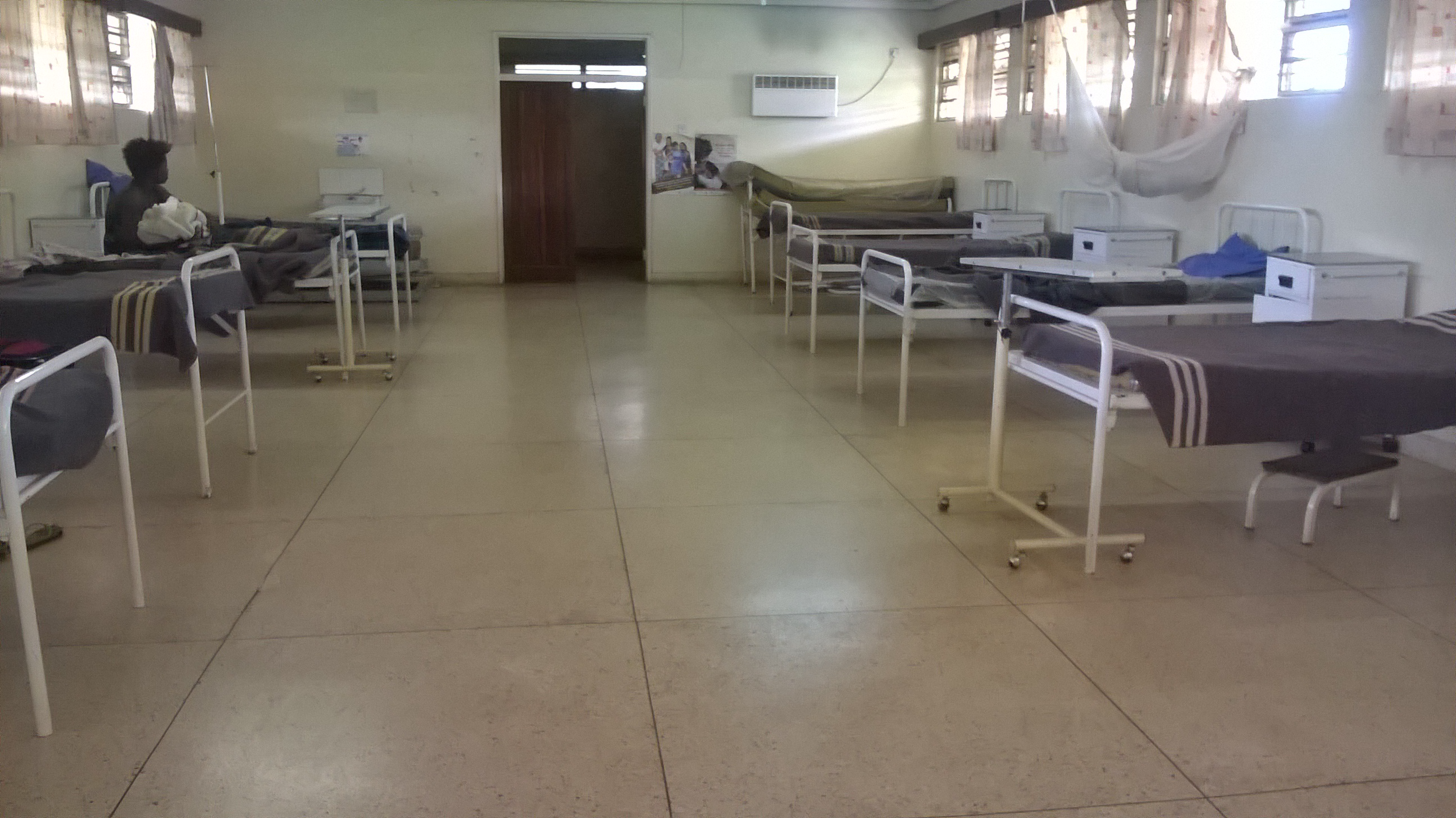  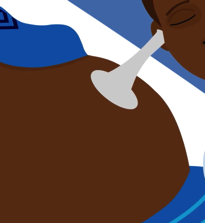  If you remain well until 37 weeks (about 8 ½ months) then your doctors will recommend delivery at this time. This means they will arrange for your labour to be induced (giving medicines to start your labour) in the same way as for women in the planned early birth group. During your monitoring, your doctors will recommend delivery before 37 weeks (about 8 ½ months) if they are concerned about either you or your baby’s condition. You will still be a part of the study if this happens.  After you have given birth, you and your baby will be cared for in the usual way at the hospital. It will not make a difference which group you belong to.  Information will be collected about yours and your baby’s health until you are both discharged from hospital.  **Do I have to take part?**  Participation in this study is entirely voluntary. You do not have to take part if you do not want to. If you decide you don't want to take part, this will not affect your care in any way. |  |  | **What are the benefits of taking part?**  The results of this study will provide additional information to improve outcomes for mothers and babies with pre-eclampsia.  We appreciate your time and are grateful for your help. However, there will not be any financial compensation for taking part in this study.  By choosing to take part in this study you will be helping us to help other women like you in the future.  **What are the possible risks of taking part?**  There are possible risks and benefits for both groups. This is why we feel it is important to do this study to improve care for women with pre-eclampsia.  If you are in the planned early birth group:  After 34 weeks (around 7 ½ months) your baby's lungs are usually mature and we know that babies who are born at this time do well. However, there is a risk that your baby may have problems associated with being born early. They may need to go to the neonatal unit when they are first born. Some of these babies may need help with their breathing or feeding in the first few days of life.  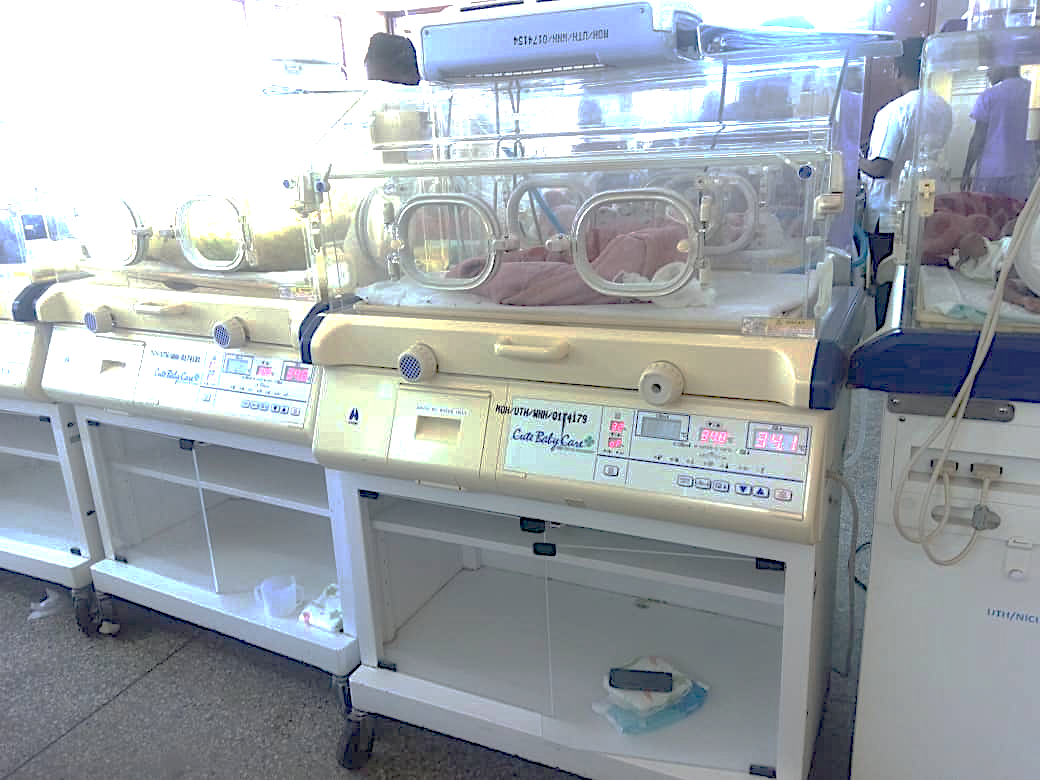 |
| --- | --- | --- | --- |
| 3 |  |  | 4 |
