# Supplementary material for: Planned early delivery versus expectant management to reduce adverse pregnancy outcomes in pre-eclampsia in a low- and middle-income setting: study protocol for a randomised controlled trial (CRADLE-4 Trial)
Source: Trials. 2020 Nov 23;21:960. doi: 10.1186/s13063-020-04888-w (PMC7684962; doi:10.1186/s13063-020-04888-w)
Supplement: Supplementary file 4 — Additional file 4. Statistical Analysis Plan. [file 13063_2020_4888_MOESM4_ESM.docx]

# CRADLE-4: Statistical Analysis Plan

| Title of clinical trial: | The CRADLE-4 Trial - Planned early delivery versus expectant management to reduce adverse pregnancy outcomes in pre-eclampsia in a low and middle-income setting. |
| --- | --- |
| Sponsor: | King's College London |
| Chief Investigator: | Professor Andrew Shennan |
| Senior Statistician: | Mr. Paul Seed |
| ISRCTN number: | ISRCTN 10672137 |
| REC number: | King's College London: HR-19/20-13535  University of Zambia: UNZA-301/2019  Bagalkot (India): SNMCIEC/1.1/2019-2020  Belagavi (India): KAHER/IEC/2019-20/D-251119016 |
| Version | 1.0 |
| Authors | Paul T Seed, Andrew Shennan, Lucy Chappell & Alice Beardmore-Gray |

Final version approved by:

| Name | Signature | Date |
| --- | --- | --- |
| Andrew Shennan | 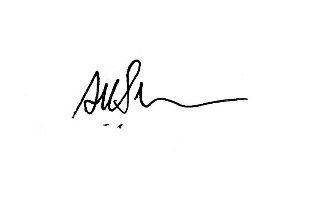 | 29.07.2020 |
| Paul Seed | **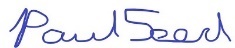** | 29.07.2020 |

Table of Contents

[CRADLE-4: Statistical Analysis Plan 1](#_Toc46921661)

[1 STUDY SYNOPSIS 4](#_Toc46921662)

[1.1 Synopsis 4](#_Toc46921663)

[2 STUDY OBJECTIVES 4](#_Toc46921664)

[2.1 Primary objectives 4](#_Toc46921665)

[2.2 Secondary objectives 4](#_Toc46921666)

[3 STUDY METHODS 5](#_Toc46921667)

[3.1 Trial design 5](#_Toc46921668)

[3.2 Setting 5](#_Toc46921669)

[3.3 Inclusion and exclusion criteria 5](#_Toc46921670)

[3.4 Comparative analysis population 6](#_Toc46921671)

[3.4.1 Short term maternal outcomes 6](#_Toc46921672)

[3.4.2 Short-term perinatal outcomes 6](#_Toc46921673)

[3.4.3 Protocol non-compliances 6](#_Toc46921674)

[3.4.4 Descriptive analysis population 7](#_Toc46921675)

[3.4.5 Post randomisation exclusions 7](#_Toc46921676)

[4 STUDY OUTCOMES 8](#_Toc46921677)

[4.1 Primary outcomes 8](#_Toc46921678)

[4.2 Secondary outcomes (maternal) 8](#_Toc46921679)

[4.3 Secondary outcomes (infant) 10](#_Toc46921680)

[5 Health resource evaluation 11](#_Toc46921681)

[6 SAMPLE SIZE 11](#_Toc46921682)

[7 STATISTICAL METHODS 12](#_Toc46921683)

[7.1 Intent-to-treat (ITT) 12](#_Toc46921684)

[7.2 Interim analyses 12](#_Toc46921685)

[7.3 Main analysis 12](#_Toc46921686)

[7.3.1 Subgroup analysis 12](#_Toc46921687)

[7.3.2 Sensitivity analysis 13](#_Toc46921688)

[7.4 Methods for dealing with missing data, unused data and false data. 13](#_Toc46921689)

[7.4.1 Missing data 13](#_Toc46921690)

[7.4.2 False data 13](#_Toc46921691)

[7.5 Software 14](#_Toc46921692)

[7.6 Statistical reporting conventions 14](#_Toc46921693)

[8 SECONDARY OBJECTIVES (Qualitative data and healthcare facilities audit) 14](#_Toc46921694)

[8.1 Qualitative data (secondary objective 3) 14](#_Toc46921695)

[8.2 Audit of facilities (secondary objective 4) 14](#_Toc46921696)

[9 REFERENCES 15](#_Toc46921697)

[10 APPENDIX 1 16](#_Toc46921698)

# [STUDY SYNOPSIS](javascript:__doPostBack()

## Synopsis

This is an individual patient randomised controlled trial which aims to establish the optimal timing of delivery in late preterm pre-eclampsia in a low and middle-income setting.

Pregnant women in India and Zambia with a confirmed diagnosis of pre-eclampsia between 34^+0^ and 36^+6^ weeks, not requiring immediate delivery, will be invited to take part. Following randomisation, they will be allocated to either planned early delivery (intervention arm) or expectant management (control arm). Maternal and infant outcome data will be collected until primary discharge from hospital.

Results will be reported according to the recommendations of the CONSORT group(1).

# [STUDY OBJECTIVES](javascript:__doPostBack()

## [Primary objective](javascript:__doPostBack()s

The aim of this trial is to establish whether planned early delivery in pre-eclampsia between 34^+0^ and 36^+6^ weeks can reduce adverse pregnancy outcomes compared to expectant management in a low and middle-income setting, without significantly increasing risk to the infant.

The primary objectives are:

1. Effectiveness: To evaluate whether planned early delivery for women with pre-eclampsia between 34^+0^ and 36^+6^ weeks of gestation can reduce maternal mortality and morbidity based on a composite of outcomes during pregnancy and delivery, until primary hospital discharge.
2. Safety: To evaluate the impact of the intervention on short term perinatal outcomes. These will be assessed based on a composite of one or more of stillbirth, neonatal death or neonatal unit admission for >48hrs due to neonatal morbidity, until primary hospital discharge.

## [Secondary objectives](javascript:__doPostBack()

The secondary objectives are:

1. To evaluate the impact of the intervention on individual components of the primary outcomes and other secondary short-term outcomes for the woman and baby

2. To evaluate the impact of the intervention on health resource use

3. To assess how the intervention influences the experiences of women and their families

4. To evaluate how the effectiveness of the intervention and its implementation is influenced by external factors - specifically resource availability and health system factors.

# [STUDY METHODS](javascript:__doPostBack()

## Trial design

This will be a pragmatic, multicentre, individual randomised controlled trial of planned early delivery versus expectant management in women with pre-eclampsia between 34^+0^ - 36^+6^ weeks’ gestation inclusive.

Following an informed consent process, trial participants will be randomised via an online database (MedSciNet) to one of two treatment arms:

Intervention arm - planned early delivery (via induction of labour or caesarean section as appropriate) within 48hrs following randomisation.

Control arm - expectant management (according to local guidelines) until either 37 weeks’ gestation is reached or an indication necessitating preterm delivery develops (as judged by the responsible clinician).

- The allocation ratio of intervention (planned early delivery) to control (expectant management) will be 1:1. Participants will be stratified by centre and minimised by parity (0 or ≥1), single/multi-fetal pregnancy (singleton or multi-fetal) and gestational age (34^+0^-34^+6^, 35^+0^-35^+6^, 36^+0^-36^+6^) at randomisation. MedSciNet will write the randomisation programme and hold the allocation code.

Women and their infants will be followed up until their primary discharge from hospital.

## Setting

The trial will be taking place across multiple urban and peri-urban sites in India and Zambia. There are central sites and referring healthcare facilities (a mixture of primary level hospitals and clinics). Trial sites will therefore comprise a mixture of CEmONC (comprehensive emergency obstetric and newborn care) and BEmONC (basic emergency obstetric and newborn care) facilities, with delivery rates ranging from 500-1000 per month at each of the central sites.

## [Inclusion](javascript:__doPostBack() and exclusion criteria

- Women who meet the following criteria will be eligible for enrolment into the study:
- Able to give valid written, informed consent
- Viable ongoing pregnancy at time of recruitment
- Clinical diagnosis of pre-eclampsia confirmed by the obstetric team: must fulfil minimum criteria of hypertension and proteinuria after 20 weeks' gestation. Hypertension will be defined as a systolic blood pressure of ≥ 140mmHg and/or a diastolic blood pressure of ≥90mmHg (or on anti-hypertensive drug at enrolment). Proteinuria will be defined as a 'positive' (≥ 1 + protein) urine dipstick result(2).
- Gestational age between 34^+0^ and 36^+6^ confirmed by a doctor (as determined by known LMP date validated by early or late ultrasound scan if available)
- Exclusion Criteria:
- Women will be ineligible if a decision to deliver within 48hrs has already been made by a senior clinician.

## Comparative analysis population

### Short term maternal outcomes

All women randomised will be included in the intention to treat (ITT) population, minus post-randomisation exclusions (see section 3.4.5).

### Short-term perinatal outcomes

Since the hypothesis being tested for these outcomes is a non-inferiority hypothesis, both an ITT and per protocol (PP) analysis will be undertaken. The per protocol population will include the babies of all mothers randomised, minus post-randomisation exclusions (see section 3.4.5), minus those randomised in error and minus those who did not receive the allocated intervention (see section 3.4.3).

### Protocol non-compliances

All protocol non compliances will be listed in the final report. Non compliances are defined below:

#### Major

The following will be defined as major protocol non-compliances:

- Data considered fraudulent

#### Minor

The following will be defined as minor protocol non-compliances:

Participants randomised in error

These include women:

- who are not between 34^+0^ and 36^+6^ weeks’ gestation inclusive
- who do not have a clinical diagnosis of pre-eclampsia as defined in the inclusion criteria
- who do not have a viable fetus
- whose consent to take part has not been fully documented
- for whom a decision has already been made to deliver within the next 48 hours

Participants who do not receive allocated intervention

These include women:

- in the ‘expectant management’ arm who received non-indicated delivery prior to 37 weeks’ gestation
- in the ‘planned immediate delivery’ arm who discontinued the intervention i.e. changed their mind after being randomised to ‘planned immediate delivery’ arm
- who were randomised to ‘planned immediate delivery’ but initiation of delivery is beyond 48 hours post-randomisation.

### Descriptive analysis population

Baseline demographic and clinical characteristics will be reported for all women randomised excluding post-randomisation exclusions (see section 3.4.5).

### Post randomisation exclusions

Exclusions to the analysis population post randomisation consist of the following:-

- Women for whom a consent form was not received
- Women for whom consent to use their data was withdrawn

(Women can specify whether data collected up to the point of withdrawal can be used. If the response is ‘No’, then they will be considered post-randomisation exclusions. If the response is ‘Yes’, then they will be reported as ‘missing’ for any data not collected after withdrawal).

- Women for whom an entire record of fraudulent data was detected

(Should fraudulent data be detected, consideration will be given to excluding all data for the site where such data was found).

The numbers (with percentages of the randomised population) of post-randomisation exclusions will be reported by randomised treatment group, and reasons summarised.

# [STUDY OUTCOMES](javascript:__doPostBack()

All outcomes are collected from trial randomisation to primary hospital discharge of the woman or infant (or 42 days post birth, whichever occurs sooner).

## [Primary](javascript:__doPostBack() outcomes

The co-primary short-term maternal outcome is:

Composite of maternal mortality and morbidity based on miniPIERS outcomes (3) with the addition of recorded systolic blood pressure ≥160mmHg (with or without medication) post randomisation. Individual components of the composite are listed in section 4.2.

The co-primary short-term perinatal outcome is:

Composite of one or more of the following: antenatal/intrapartum stillbirth or neonatal death (but not deaths due to congenital anomalies) or neonatal unit admission >48hrs due to neonatal morbidity (as defined by an indication for admission to the neonatal unit according to local tertiary hospital neonatal guidelines, provided in Appendix 1) until primary hospital discharge. Each neonate will be considered separately, with no double counting of outcomes.

## Secondary outcomes (maternal)

Secondary short-term maternal outcomes

|  | Component of primary outcome | Component detected by a clinical diagnosis | Component detected using additional resources with variable availability | Tested |
| --- | --- | --- | --- | --- |
| Number of women with maternal morbidity and mortality component of the primary outcome | Yes | N/A | N/A | Yes |
| Number of women with severe hypertension component of the primary outcome | Yes | N/A | N/A | Yes |
| Number of women with maternal morbidity and mortality composite of components detected by a clinical diagnosis only | Yes | Yes | No | Yes |
| **Individual components of the primary outcome (non-exclusive):** |  |  |  |  |
| Maternal death | Yes | Yes | No | No |
| Hepatic hematoma or rupture | Yes | Yes | No | No |
| Glasgow coma score <13 | Yes | Yes | No | No |
| Stroke | Yes | Yes | No | No |
| Cortical blindness | Yes | Yes | No | No |
| Reversible ischaemic neurological deficit | Yes | Yes | No | No |
| Retinal detachment | Yes | Yes | No | No |
| Postpartum haemorrhage requiring transfusion or hysterectomy | Yes | Yes | No | Yes |
| Placental abruption | Yes | Yes | No | Yes |
| Myocardial ischaemia/infarction | Yes | Yes | No | No |
| Eclampsia | Yes | Yes | No | Yes |
| Require >50% oxygen for greater than one hour | Yes | Yes | No | No |
| Severe breathing difficulty^ⱡ^ | Yes | Yes | No | No |
| Pulmonary oedema | Yes | Yes | No | No |
| Hepatic dysfunction | Yes | No | Yes | Yes* |
| Acute renal insufficiency | Yes | No | Yes | Yes* |
| Dialysis | Yes | No | Yes | No |
| Transfusion of blood products | Yes | No | Yes | No |
| Platelet count <50, 000 without blood transfusion | Yes | No | Yes | Yes* |
| Positive inotropic support | Yes | No | Yes | No |
| Intubation other than for caesarean section | Yes | No | Yes | No |
| **Additional secondary outcomes:** |  |  |  |  |
| Length of stay in hospital (prior to delivery and after delivery) | No | N/A | N/A | Yes |
| Time from randomisation to delivery (process outcome) | No | N/A | N/A | Yes |
| Intensive care unit admission | No | N/A | N/A | No |
| Use of Magnesium Sulfate | No | N/A | N/A | No |
| Use of Antenatal Corticosteroids | No | N/A | N/A | No |
| Use of Antihypertensives | No | N/A | N/A | No |
| Time from randomisation to initiation of delivery | No | N/A | N/A | No |
| Mode of onset of birth (spontaneous, induced or pre-labour caesarean section) | No | NA | NA | No |
| Primary indication for delivery in both arms | No | NA | NA | No |
| Serious adverse events | No | NA | NA | No |

* These will only be tested if missing data is <20% in both arms

## Secondary outcomes (infant)

Tested:

- Individual components of the primary outcome:
- Stillbirth
- Neonatal death before primary hospital discharge*
- Neonatal unit admission >48hrs due to neonatal morbidity

*Subject to a minimum pooled event rate of 5%.

- Mode of delivery (vaginal vs. all others)
- Respiratory support required
- Supplementary oxygen required
- Median gestational age at delivery
- Birthweight centile
- Birthweight centile less than tenth centile
- Admissions to neonatal unit
- Number of nights in neonatal unit (acute and sub-acute level of care) for babies admitted.
- APGAR score at 5 minutes post birth
- Neonatal resuscitation required

Not tested:

- Delivery before 37 weeks
- Median Birthweight (kg)
- Birthweight centile less than the third centile
- Hypoglycaemia requiring intervention
- Respiratory Distress Syndrome (RDS)
- Supplementary oxygen (Yes/No and duration)
- Continuous positive airways pressure (Yes/No and duration)
- Invasive ventilation support (Yes/No and duration)
- Primary indication for neonatal unit admission
- Sepsis - with evidence of confirmed infection
- Course of antibiotics given for Possible Serious Bacterial Infection (according to WHO's Integrated Management of Childhood Illness (IMCI) guidelines)
- Apgar score at 10 minutes post birth
- Hypoxic Ischaemic Encephalopathy and Grade
- Administration of surfactant
- Diagnosis of necrotising enterocolitis (diagnosed at surgery or resulting in death)
- Neonatal seizures requiring anti-convulsants
- Nasogastric feeding required and indication
- Hypothermia (Temperature <36.5 degrees Celsius)
- Neonatal jaundice requiring phototherapy
- Exclusively breast fed at discharge
- Serious adverse events

# Health resource evaluation

A health economics analysis plan will be provided separately.

# [SAMPLE](javascript:__doPostBack() SIZE

Each country will use the same intervention and work to the same protocol.  The sample size for the CRADLE 4 study is calculated on the ability to detect a clinically important reduction in the primary maternal outcome: a short-term composite based on the presence of one or more of 22 maternal morbidities.

- Based on the data available from the CRADLE-4 Phase 1 Feasibility Study we anticipate an event rate of 80% in the expectant management arm. We have calculated that a sample size of 558 would provide 90% power to detect a 15% relative risk reduction. If the trial is recruiting well, we will continue to recruit 872 participants which would give 90% power to detect a 12.5% relative risk reduction and greater precision to detect secondary outcomes. The Data Monitoring Committee (DMC) will review the primary event rate and usual safety data and make a recommendation to continue or stop.

| - Relative risk reduction | - Event rate (expectant management) | - Event rate - (planned delivery) | - Sample size (women with complete data) | - Recruitment target allowing for up to 10% loss to follow up |
| --- | --- | --- | --- | --- |
| - 15% | - 80% | - 68% | - 558 | - 620 |
| - 12.5% | - 80% | - 70% | - 784 | - 872 |

- A one-sided non-inferiority analysis is planned for the primary perinatal composite. Our Phase 1 data suggests 54 neonatal events out of 234 deliveries (24.35%). Complete data on 480 women (240 per group) are required for 90% power to exclude a difference against planned delivery of 10% or more. To exclude a difference of 7.5%, 852 women (426 per group) are needed. The calculation uses a one-sided significance test and confidence interval and assumes that the true event rate is 24%, as in the Phase 1 Study. This is in line with the planned sample size as detailed above. There will be an additional analysis (subject to approval by the Trial Steering Committee) to test efficacy of the intervention on the primary perinatal outcome.

# STATISTICAL METHODS

## [Intent-to-treat (ITT)](javascript:__doPostBack()

All analyses will be based on the intention-to-treat (ITT) principle, except as described in section 3.4.

## Interim analyses

No formal interim analysis is planned. The Data Monitoring Committee (DMC) will review the primary event rate and usual safety data and make a recommendation to continue or stop. Stopping for efficacy will be based on the Haybittle-Peto principle that overwhelming evidence is needed in favour of one treatment option such that randomisation is no longer ethical.

## Main analysis

All outcomes will be analysed adjusting for minimisation factors. Binary outcomes will be analysed using log binomial regression models. Results will be presented as adjusted risk ratios with associated confidence intervals (CI). If the model does not converge, logistic regression with robust variance estimation will be used. Continuous outcomes will be analysed using linear regression models. Results will be presented as differences in means with associated CIs. 95% CIs will be presented for all primary outcomes and 99% CIs for secondary outcomes.

For the analysis of perinatal outcomes, we will treat all infants (singletons or multiples) separately, adjusting standard errors for clustering by mother. Loss to follow-up is expected to be about 5% for the short-term outcomes.

A secondary per-protocol analysis will look at the primary perinatal outcomes according to the treatment actually received and time of randomisation (see section 3.4 for per-protocol population).

The primary maternal outcome will be reported as a composite of maternal morbidity and mortality, and severe hypertension. Individual components of the maternal morbidity composite will be further divided and reported separately as a maternal morbidity and mortality composite of components detected by a clinical diagnosis only.

### Subgroup analysis

Pre-specified subgroup analyses will be undertaken for gestation at randomisation (test for trend) and for single vs. multi-fetal pregnancy, country and region (with a region being tertiary centre and referring healthcare facilities). The consistency of the effect of planned delivery vs. expectant management across subgroups will be assessed using a likelihood ratio test for interaction.

### Sensitivity analysis

A sensitivity analysis for the co-primary outcomes (maternal and perinatal) in those women whose delivery was initiated within 96hrs will be performed.

## Methods for dealing with missing data, unused data and false data.

### Missing data

We will follow a four-point framework for dealing with incomplete observations which will allow the correct method to be chosen and subsequently implemented(4).

1. Attempt to follow up all randomised participants, even if they withdraw from allocated treatment
2. Perform a main analysis of all observed data that is valid under a plausible assumption about the missing data. Specifically, we will assume data is missing at random (MAR). Under this assumption, imbalances between treatment groups due to dropout can be corrected by appropriate multiple regression models.
3. Perform a sensitivity analyses to explore the effect of departures from the assumption made in the main analysis. The MNAR (missing not at random) analysis will use the method of White et al.(4) as implemented in the Stata command rctmiss.
4. Account for all randomised participants, at least in the sensitivity analyses

This framework highlights the importance of using plausible assumptions with regards to the nature of the missing data. These assumptions will then be tested using appropriate sensitivity analyses on observed data using complete case analysis. For the purpose of the main analysis we will make the assumption that missing data is missing at random and the effect of the intervention is the same in those with and without the observations.

Furthermore, we will check whether or not there is an imbalance in the percentage of missing data within each treatment allocation.

### False data

We will take all reasonable precautions to minimise the number of data errors. Everyone responsible for collecting data will be trained in the procedures to follow, as laid down in the trial protocol and handbook. All data entered will be checked by the trial coordinator when entered on the data base and again by the statistician at the time of analysis. Corrections will be made wherever possible. Fraudulent data is discussed in section 3.4.

## [Software](javascript:__doPostBack()

Analyses will be performed using Stata Version 16 or later (StataCorp, College Station, Texas, USA).

## Statistical reporting conventions

Rounding: percentages will be presented to the nearest whole number. Averages, SD etc. of continuous measures will be given to 2 or (where appropriate) 3 significant figures.

Arithmetic means (SD) presented for continuous variables which are approximately normally distributed; geometric means (SD) for log-Normal distributions. Medians (quartiles) otherwise.

Comparisons between treatment groups will be presented with 95% Confidence intervals, and standard errors.

P-values will be given to 2 significant figures or 3 decimal places, except where <0.0001 is appropriate. 95% Confidence Intervals used, and conventional significance will be taken at P<0.05.

# SECONDARY OBJECTIVES (Qualitative data and healthcare facilities audit)

## Qualitative data (secondary objective 3)

To assess how the intervention influences the experiences of women and their families:

- We will conduct in depth patient interviews with a purposeful sample of trial participants
- The qualitative data will be analysed using NVivo software and a thematic framework approach.

## Audit of facilities (secondary objective 4)

To evaluate how the effectiveness of the intervention and its implementation is influenced by resource availability and external factors affecting the performance of the health system

- We will conduct facility level resources audit every 6 months during the trial
- The audit data will be reported using descriptive statistics only

# REFERENCES

1. The Lancet. Consort 2010. Lancet. 2010;375(9721):1136.

2. Brown MA, Magee LA, Kenny LC, Karumanchi SA, McCarthy FP, Saito S, et al. Hypertensive disorders of pregnancy: ISSHP classification, diagnosis, and management recommendations for international practice. Hypertension. 2018;72(1):24–43.

3. Payne BA, Hutcheon JA, Ansermino JM, Hall DR, Bhutta ZA, Bhutta SZ, et al. A Risk Prediction Model for the Assessment and Triage of Women with Hypertensive Disorders of Pregnancy in Low-Resourced Settings: The miniPIERS (Pre-eclampsia Integrated Estimate of RiSk) Multi-country Prospective Cohort Study. PLoS Med. 2014;11(1).

4. White IR, Horton NJ, Carpenter J, Pocock SJ. Strategy for intention to treat analysis in randomised trials with missing outcome data. Bmj. 2011;342(7803):910–2.

# APPENDIX 1

Indications for admission to neonatal intensive care unit

- Weight less than 1.8kg
- In respiratory distress
- Temperature greater than 38 degrees Celsius
- Hypo-glycaemia unresponsive to feeds
- Macrosomic baby above 4kg
- All infants of diabetic mums
- Infants with meconium aspiration
- Infants with congenital anomalies
- Asphyxiated babies
- Babies with convulsions
- Babies with Jaundice
- Persistent vomiting
- All infants with a septic risk (PROM >18 hours, maternal UTI etc.)
- Hypothermia (temperature <36 degrees Celsius) unresponsive to warming by radiant warmer/KMC)

(according to the Neonatal Protocols of University Teaching Hospital, Lusaka, Zambia and approved by co-investigators at KLE Academy of Higher Education and Research, JNMC, Belagavi, Karnataka, India)
